# Multidrug resistance among *P. aeruginosa *and Enterobacteriaceae in US hospitals, 2000 to 2009

**DOI:** 10.1186/cc12013

**Published:** 2013-03-19

**Authors:** M Zilberberg, A Shorr

**Affiliations:** 1 EviMed Research Group, LLC, Goshen, MA, USA; 2 Washington Hospital Center, Washington, DC, USA

## Introduction

Gram-negative resistance remains a major challenge in the care of critically ill patients. Traditionally, *P. aeruginosa *(PA) has represented the most concerning pathogen. However, carbapenemase-producing Enterobacteriaceae (CPE) have emerged as a challenge, but the epidemiology of this pathogen is poorly understood.

## Methods

We analyzed a large US-based microbiology database, Eurofins TSN, between the years 2000 and 2009. We aimed to describe the prevalence of infection with either multidrug-resistant (MDR) PA or CPE. We defined MDR-PA as any PA isolate resistant to ≥3 drug classes. Enterobacteriaceae were classified as CPE if resistant to both a third-generation cephalosporin and a carbapenem. We evaluated specimens from respiratory, bloodstream, urinary tract (UTI) and complicated intra-abdominal infections.

## Results

We identified 327,912 PA (60,695 (18.5%) MDR-PA) and 279,600 Enterobacteriaceae (2,558 (0.9%) CPE) specimens. More than one-quarter (26.1%) of all PA were recovered from ICU patients as compared with 17.9% of all Enterobacteriaceae specimens. Of those specimens originating in the ICU, MDR-PA represented 21.9% of all PA organisms, while CPE represented 1.6% of all Enterobacteriaceae. Pneumonia and UTI accounted for 92.4% of all PA and 84.0% of all Enterobacteriaceae specimens. The proportion of both MDR-PA and CPE was highest in pneumonia (22.0% and 1.6%, respectively) and lowest in UTI (13.7% and 0.6%, respectively). Over the time frame of the study, CPEs emerged and stabilized at approximately 2.8% of all Enterobacteriaceae, while MDR-PA increased slightly from 16.0% of all PA in 2000 to 17.8% in 2009.

## Conclusion

Although CPE organisms have emerged as an important pathogen, MDR-PA remains an order of magnitude more prevalent in the United States. Pneumonia patients and those in the ICU are at an increased risk for both MDR-PA and CPE infections compared with those outside the ICU.

